# Lifestyle Medicine and Economics: A Proposal for Research Priorities Informed by a Case Series of Disease Reversal

**DOI:** 10.3390/ijerph182111364

**Published:** 2021-10-29

**Authors:** Kara A. Livingston, Kelly J. Freeman, Susan M. Friedman, Ron W. Stout, Liana S. Lianov, David Drozek, Jamie Shallow, Dexter Shurney, Padmaja M. Patel, Thomas M. Campbell, Kaitlyn R. Pauly, Kathryn J. Pollard, Micaela C. Karlsen

**Affiliations:** 1 American College of Lifestyle Medicine, Chesterfield, MO 63006, USA; kara.livingston@gmail.com (K.A.L.); globalpositivehealth@gmail.com (L.S.L.); drozek@ohio.edu (D.D.); dshurney@gmail.com (D.S.); padmaja.patel@midlandhealth.org (P.M.P.); 2 Department of Member Engagement & Administration, American College of Lifestyle Medicine, Chesterfield, MO 63006, USA; kfreeman@lifestylemedicine.org (K.J.F.); kpauly@lifestylemedicine.org (K.R.P.); 3 School of Nursing, Indiana University, Indianapolis, IN 46202, USA; 4 School of Medicine and Dentistry, University of Rochester, Rochester, NY 14620, USA; susan_friedman@urmc.rochester.edu; 5 Ardmore Institute of Health, Ardmore, OK 73401, USA; ron.stout@ardmoreinstituteofhealth.org; 6 Global Positive Health Institute, Sacramento, CA 95825, USA; 7 Department of Specialty Medicine, Heritage College of Osteopathic Medicine, Ohio University, Athens, OH 45701, USA; 8 Bright Health Group, Minneapolis, MN 55437, USA; jshallow@brighthealthgroup.com; 9 BlueZones Well-Being Institute, Adventist Health, Roseville, CA 95661, USA; 10 Midland Health, Midland, TX 79703, USA; 11 Medical Center, University of Rochester, Rochester, NY 14642, USA; thomas_campbell@urmc.rochester.edu; 12 Department of Research, American College of Lifestyle Medicine, Chesterfield, MO 63006, USA; kpollard@lifestylemedicine.org

**Keywords:** lifestyle medicine, diabetes remission, chronic disease, healthcare costs, cost savings

## Abstract

Chronic disease places an enormous economic burden on both individuals and the healthcare system, and existing fee-for-service models of healthcare prioritize symptom management, medications, and procedures over treating the root causes of disease through changing health behaviors. Value-based care is gaining traction, and there is a need for value-based care models that achieve the quadruple aim of (1) improved population health, (2) enhanced patient experience, (3) reduced healthcare costs, and (4) improved work life and decreased burnout of healthcare providers. Lifestyle medicine (LM) has the potential to achieve these four aims, including promoting health and wellness and reducing healthcare costs; however, the economic outcomes of LM approaches need to be better quantified in research. This paper demonstrates proof of concept by detailing four cases that utilized an intensive, therapeutic lifestyle intervention change (ITLC) to dramatically reverse disease and reduce healthcare costs. In addition, priorities for lifestyle medicine economic research related to the components of quadruple aim are proposed, including conducting rigorously designed research studies to adequately measure the effects of ITLC interventions, modeling the potential economic cost savings enabled by health improvements following lifestyle interventions as compared to usual disease progression and management, and examining the effects of lifestyle medicine implementation upon different payment models.

## 1. Introduction

Chronic disease places an enormous economic burden on both individuals and the healthcare system. Existing fee-for-service models of healthcare prioritize symptom management, medications, and procedures over treating the root causes of disease through changing health behaviors [1]. The U.S. health system faces major challenges to contain the substantially increasing healthcare costs as the fee-for-service approach to care tends to incentivize increased healthcare spending [2]. Despite attempts from multiple reimbursement models, such as accountable care organizations (ACOs) [1,3] and risk-based reimbursement [4] to contain costs and improve outcomes, it is urgent to prevent and reverse the conditions driving the costs, namely, the poor health habits [5,6,7,8] contributing to rising rates of chronic disease [9]. Accumulated chronic and mental health conditions are responsible for 90% of the USD 3.8 trillion annually spent on healthcare in the U.S. [10].

Projections for prevalence and financial burden of diabetes, atherosclerotic cardiovascular disease (ASCVD), and obesity are grim. By 2030, the prevalence of type 1 and type 2 diabetes is estimated to increase by 54%, with total annual societal and medical costs of USD 622 billion [11]. The prevalence of CVD is estimated to increase to 41% with annual total direct medical costs of USD 818 billion [12], and the prevalence of obesity is expected to rise to approximately 50% of adults [13], with resulting loss of economic productivity of up to USD 580 billion annually [14]. Cumulatively, almost 370 billion dollars is spent annually for retail drug prescriptions alone [15]. 

Along with a lower quality of life and increased mortality, individual costs of poor health are substantial [16]. The effects of non-communicable chronic diseases along with their associated medical costs have been cited as a frequent cause of bankruptcy in the United States [16,17]. Recent National Health Interview Survey Data found that among adults with diabetes, 41% reported financial hardship due to their medical bills [18]. Individuals with diagnosed type 2 diabetes are met with approximately USD 16,750 per year in medical expenditures, with approximately USD 9600 of this cost directly related to the diabetes [19]. Atherosclerotic cardiovascular disease (ASCVD) is thought to be the most expensive chronic disease not only from a total healthcare cost standpoint, but also for individual costs, which tend to be over USD 2000 annually out-of-pocket per individual with insurance [20]. Adults with obesity generate an additional USD 3508 per individual annually in total healthcare costs [21]. 

However, many chronic conditions can be prevented, treated and improved, or even reversed through lifestyle modifications, particularly a transition to a whole food, plant-based (WFPB) diet [22,23,24,25,26,27,28,29,30,31,32,33,34,35]. Research has shown that plant-based diets, specifically WFPB diets, promote weight loss and healthy weight maintenance, decrease risk of type 2 diabetes, and reduce CVD risk factors, including lowering triglycerides and biomarkers of CVD, effectively reducing hypertension, insulin resistance, and oxidative stress [22,36,37,38,39,40,41,42,43,44,45,46,47,48,49]. Research has additionally shown that diet and environment may play a role in autoimmune disease, with Western diets potentially increasing inflammation and insulin resistance [50,51,52,53,54,55]. 

Thus, lifestyle medicine (LM) offers enormous potential to both promote health and reduce healthcare costs, though such economic outcomes have yet to be quantified. LM is the use of evidence-based lifestyle therapeutic intervention—including a whole-food, plant-predominant eating pattern, regular physical activity, restorative sleep, stress management, avoidance of risky substances, and positive social connection—as a primary modality, delivered by clinicians trained and certified in this specialty to prevent, treat, and often reverse chronic disease [56]. 

As value-based care gains traction and becomes competitive with the fee-for-service approach [57], a need emerges for value-based care models that also achieve the quadruple aim [58] of (1) improved population health, (2) enhanced patient experience, (3) reduced healthcare costs, and (4) improved work life and decreased burnout of healthcare providers. In addition, in 2010, the Affordable Care Act (ACA) authorized the Center for Medicare and Medicaid Innovation (CMMI) “…to test innovative payment and service delivery models to reduce program expenditures…while preserving or enhancing the quality of care furnished to individuals.” However, a recent review [57] suggested that of over 50 models tested, only 5 demonstrated “significant financial savings.” As far as the authors are aware, none of these models have specifically included robust lifestyle medicine programming. Thus, there is significant, untapped potential for CMMI and other stakeholders to transform healthcare finance and delivery [59].

The first objective of this paper to demonstrate a proof of concept of healthcare cost savings following intensive, therapeutic lifestyle interventions, utilizing four individual case studies. The second objective is to propose priorities for lifestyle medicine economic research related to the components of quadruple aim—improved population health, improved patient experience, reduced healthcare costs, and provider well-being [58]. 

## 2. Materials and Methods

Four individual cases of disease reversal and associated changes in healthcare costs were identified—two cases through referral and personal introduction to the research team and two cases through email marketing to the newsletter list of the American College of Lifestyle Medicine (ACLM). All data were self-reported by individual cases, and case histories were collected using an online survey hosted in QuestionPro in the spring of 2021. Questions asked followed the timeline of their previous diagnosis, lifestyle changes, and cost details for their diet, lifestyle, and healthcare, both pre- and post-lifestyle change. Participants were asked to provide clarifications and follow-up as necessary via email. Participants reviewed this manuscript prior to publication and provided consent for inclusion. 

## 3. Results: Case Series of Disease Reversal Following Intensive Lifestyle Modification

### 3.1. Case 1

#### 3.1.1. Overview of Condition

Case 1 is a 47-year-old male who recalls gradually worsening illness starting around the age of 25 that he partially attributes to poor food choices and opioid use. By the age of 36, he recalls knowing that he had “undeniable, serious illness,” and by the age of 38, he weighed over 400 pounds and suffered from severe sleep apnea, asthma and trouble breathing, eczema, allergies, hypertension (SBP/DBP = 255/155 on 2 medications), high total cholesterol (300 mg/dL), high triglycerides (279 mg/dL), and chronic shoulder and joint pain. He had difficulty moving and completing basic activities of daily living, such as putting on his shoes. He was on a number of medications that cost him approximately USD 7680 out of pocket, annually, while his insurance company paid a total of USD 19,646 for medications in 2009 alone. In addition to seeing a physician at least once every 1–2 weeks for his various conditions, he attended monthly appointments to aspirate fluid from his knees, had multiple visits to the ER, underwent shoulder surgery, and had one ankle reconstruction surgery with a second one imminent. Around 2012, at the age of 38, he decided to pursue gastric bypass surgery; however, his physician did not clear him to undergo the procedure, due to his poor underlying health. This event, combined with the recent deaths of two close family members, is what ultimately prompted him to begin considering lifestyle changes to improve his health. Initially, his approach to change was “going one step further than the day before.” First, he forced himself to simply get up from his chair once, and then twice per day, and then he progressed to being able to go on walks and walk up the stairs. He began researching additional lifestyle changes and watched documentaries, such as *Forks Over Knives*, which he describes as providing an entirely new perspective on food. He adopted dietary changes that began with a green juice fast followed by calorie restrictions, reducing red meat, and changing from full to lower fat dairy. On 1 January 2013, he adopted a strict, whole-food, plant-based (WFPB) dietary pattern. Thus, what started initially as a set of small lifestyle changes ultimately fostered a completely new lifestyle approach—one where fitness and plant-based eating were the focus. Approximately 6 months into the lifestyle change, Case 1 had lost 150–200 pounds, and although he does not recall his exact numbers, his cholesterol was cut in half, and triglyceride levels were in the normal range. By 2015, his cholesterol was 112 mg/dL and triglycerides were 74 mg/dL. In just 3–4 years, he went from barely standing up from a chair to running his first marathon in May 2016; by 2017, he was completing Ironman competitions and later ran an ultra-marathon. His current laboratory values as of 20 May 2021 include a total cholesterol of 103 mg/dL, HDL cholesterol 36 mg/dL, triglycerides 61 mg/dL, non-HDL cholesterol 67 mg/dL, and blood pressure 122/78. Post lifestyle change, he also notes significant improvement in his level of happiness and quality of life, specifically with respect to being able to enjoy time with his family, traveling, and gaining a new outlook on life.

#### 3.1.2. Cost Savings

Aside from the possibility of gastric bypass surgery (USD 18,000–35,000) [60], Case 1 was a candidate for a Continuous Positive Airway Pressure (CPAP) machine for obstructive sleep apnea (USD 1000–3000) [61] and a Transcutaneous Electrical Nerve Stimulator (TENS) machine for pain management (approximately USD 100–500) [62] prior to his lifestyle change, highlighting an approximate average savings of USD 28,800 in discreet medical costs potentially avoided. Post lifestyle change, he reports spending only USD 61 per year on medications (B12 injections), as opposed to his previous approximated average spending of USD 7600+ in annual medication costs. Pharmaceutical costs covered by his insurance company decreased from USD 19,000+ in 2009 to USD 122.24 in 2015 and 2016 combined. In addition, his grocery costs are approximately half of what they were prior to his lifestyle change.

### 3.2. Case 2

#### 3.2.1. Overview of Condition

Case 2 is a 49-year-old female diagnosed with type 2 diabetes (T2D) on 3 January 2020. At this time, her fasting blood glucose was measured at 266 mg/dL, and HbA1c was 10.9%. She conducted daily glucose monitoring, was prescribed Metformin for glucose control and described herself as “living on pain meds” for headaches and joint pain. She began the Kaiser Medical Weight Management Program at a starting weight of 205 lbs on 8 January 2020, which entailed medically supervised weight loss, using low-calorie meal replacements providing 960 calories per day for 15 weeks. After this initial period, she was slowly weaned off meal replacements. By 17 August 2020, she had lost 57.2 pounds, reaching her goal weight of 147.8. The Kaiser program also incorporated weekly group sessions to encourage adherence, behavioral skills to develop long-term health-promoting habits, and long-term weekly support groups. She took Metformin only from 3 January 2020 through 15 January 2020 and then discontinued it. She also adhered to a predominantly plant-based diet, including whole grains as well as some dairy, salt, Rx Bars, Clif Whey Protein Bars, and occasionally honey. Having previously completed this program in 2010, she knew that it worked, but she noted that this second time, she was motivated by her diabetes diagnosis and had significant support from her primary care physician. By April of 2020, her fasting blood glucose had dropped to 117 mg/dL and her HbA1c had dropped to 5.7%. By summer of 2020, she reported that her pain and headaches had improved, with an overall improvement in quality of life, as she was able to move around and exercise without pain or discomfort. In July of 2021, her fasting blood glucose was measured at 108 mg/dL and HbA1c was at 5.4%: she is no longer on any diabetes medications and meets the definition of diabetes remission [63].

#### 3.2.2. Cost Savings

Case 2 had low personal cost of medical care, a single USD 10 copay for Metformin, due to the fact that she pursued a major lifestyle change within less than 2 weeks of being diagnosed with T2D. The Kaiser program initially cost USD 4700 out-of-pocket and now allows her to access weekly check-ins and groups for free to encourage maintenance. She notes the opportunity cost of substantially decreased grocery bills since adhering to her healthier diet, as well as no longer eating out, which results in additional savings. 

### 3.3. Case 3

#### 3.3.1. Overview of Condition

Case 3 is a 50-year-old male who reported suffering from severe edema, hypokalemia, pre-diabetes, fatty liver, high blood pressure, obesity, severe arthritis, and “pinched nerves”. He had frequent clinician visits, as well as occasional ER and urgent care visits. He also required physical therapy and chiropractic care, his physician recommended gastric bypass surgery, and he was taking various medications as detailed in Table 1. In October 2016, unsatisfied with his current care and following a hospitalization due to severe abdominal pain, he committed to a major lifestyle change by moving toward a more plant-based diet and eliminating meat. In three months, he lost 80 lbs, lowered his blood pressure, and eliminated his edema, sleep apnea, and pre-diabetes. After watching *Forks Over Knives*, he additionally eliminated dairy and eggs from his diet. Since adhering to a plant-based diet, he reports losing 168 lbs over the course of about 20 months, from a starting weight of 348 lbs to an end weight of 180 lbs, being in excellent health, and reversing all medical conditions with the exception of his arthritis. He has normal BMI, excellent blood pressure, no remaining fatty liver or edema, and minimal joint pain. He notes a major improvement in his level of happiness and quality of life, as he is able to participate in outdoor activities that he loves, such as kayaking and biking.

#### 3.3.2. Cost Savings

He reports that his grocery bill is half of what it used to be and notes that eating beans, rice, and produce, particularly from local farms and grocers, is less expensive than his previous diet that incorporated foods, such as meats and cheeses. Specifically, he currently spends about USD 350–400/month on groceries, in comparison to the USD 700/month that he previously spent. In addition, his overall spending has decreased because he eats out very rarely. He currently spends approximately USD 150 annually to participate in plant-based educational programming. With respect to additional costs, the following interventions had been recommended prior to his lifestyle change (with various estimated costs reported for each): gastric bypass surgery (USD 18,000–35,000) [60], CPAP machine (USD 1000–3000) [61], compression stockings (USD 64–228) [64] and bilateral knee replacements (USD 58,670–68,100) [65]. However, none of these remained necessary following his intensive lifestyle change, representing an approximate average of USD 92,031 in potentially avoided discrete medical costs. Finally, Case 3 reports spending USD 486 out of pocket annually on medications prior to lifestyle change, and this cost was reduced to USD 0 post change.

### 3.4. Case 4

#### 3.4.1. Overview of Condition

Case 4 is currently a 60-year-old female who, prior to 2009, was experiencing high blood pressure, metabolic syndrome, constant migraines, frequent urinary tract infections (UTIs) or interstitial cystitis, frequent illnesses (cold, flu, etc.), rosacea, fatty liver syndrome, and hypothyroidism. She reports being on a number of medications detailed in Table 1. Although she felt satisfied with her medical care at the time, she believed that it was never getting to the root cause of her illness. In 2009, during a trip to the hospital suffering from high fever, ‘deadly’ high blood pressure, and an infection, at the suggestion of an MD/RD clinician she met in the hospital, she read Dr. Caldwell Esselstyn’s book *Prevent and Reverse Heart Disease*. After reading this book, she followed the recommended protocol and adopted a WFPB to which she has continued to adhere. She notes that her husband joined her in this dietary change and has reversed multiple sclerosis. Adopting this eating pattern led her to lose 110 lbs with a starting weight of 272 lbs, over approximately 1.5 years. She was able to discontinue all medications. In addition, she has attended plant-based summits and earned a continuing adult education plant-based nutrition certificate.

#### 3.4.2. Cost Savings

She reports that following her WFPB diet and incorporating organic foods costs about USD 1000/month (she reported USD 200–300/week) for herself, her husband, and any cooking demos she now makes. Currently, she reports having only four physician visits per year, which amount to USD 375/visit. This is in contrast to the USD 30,000 bill she received for the single ER visit and subsequent hospital stay that prompted her WFPB lifestyle. This bill was the total amount charged by the hospital before insurance payments; she does not remember the total amount she ended up paying out-of-pocket toward this. She noted that despite this huge medical bill, she went home from the hospital still experiencing chronic UTIs, trips to the doctor’s office (USD 50 each, plus additional co-pays for testing and medications), and remained on medications. Her health insurance coverage was approximately USD 35,000 annually, which was paid by her husband’s employer at the time and had a USD 4000 deductible. Various testing expenses, such as a USD 4500 colonoscopy, counted toward the deductible before any insurance payments, were made. She estimates that co-pays for high blood pressure and thyroid medications were about USD 75/month. In contrast, her current health is excellent and vibrant, and she has no medication costs, aside from vitamin B12 and vitamin D supplements. Her current insurance plan has a USD 12,000 deductible and costs USD 500/month for her husband to be included; her employer pays for her insurance. She reports being grateful for her lifestyle, which she feels enables her to avoid medical care/testing expenses, such as those prior to her lifestyle change.

## 4. Discussion

The objective of the quadruple aim is to optimize health system performance through improved population health, improved patient experience, reduced healthcare costs, and provider well-being [58]. This case series illustrates the effectiveness of intensive, therapeutic lifestyle change (ITLC), utilizing a predominately WFPB diet, in many cases coupled with physical activity, to promote the remission of chronic disease and decrease the need for medication, ultimately supporting all four components of the quadruple aim. 

With respect to health and patient experience, the outcomes in the four cases presented are far superior to the typical prognosis of these chronic conditions, which often involves increasing medical regimes and polypharmacy, increased side effects, and no prospect of health restoration. The results of this case series are consistent with previous and emerging research demonstrating that ITLC is effective in delivering significant health benefits [66], including weight loss [41,67], improvements in blood glucose among patients with T2D [68,69,70], regression of atherosclerosis [30,71,72], and even remission of autoimmune disease [73]. Beyond the dramatic physical health benefits, it is also clear that these patients have experienced increased levels of happiness and improved quality of life.

This case series provides proof of concept for the power of ITLC in driving disease remission and supports future research on the potential economic benefits of a LM approach due to cost avoidance. At minimum, Case 1 avoided two pieces of costly medical machinery (CPAP and TENS machine), at least 10 medications, a gastric bypass surgery and a second ankle reconstruction surgery. These cost savings are in addition to numerous doctor’s office and ER visits, which would likely have only continued to become more frequent as his chronic conditions progressed. Case 3′s lifestyle changes similarly brought about enormous cost savings, including avoided surgical procedures (gastric bypass, knee replacements), polypharmacy costs, and medical equipment costs. Case 3′s story also highlights significant cost savings beyond healthcare by cutting his monthly grocery bill in half. Case 4′s story is noteworthy because it highlights that, even despite a USD 30,000 bill for an ER visit and subsequent hospital stay due to her chronic illnesses, she went home with no improvement of her conditions and continued to accrue additional costs for multiple follow-up trips to the doctor’s office which were burdensome, due to her high insurance deductible.

With respect to Case 2, her medical costs as well as burden on the healthcare system remained relatively low, due to the fact that she pursued a major lifestyle change within 2 weeks of being diagnosed with diabetes. Type 2 diabetes remission, though now known to be possible for many patients [74], is a relatively unusual outcome for most individuals because they do not pursue lifestyle changes that would enable it [75]. The typical progression from a diagnosis of T2D is increasing complications, ongoing treatment, and lifetime dependence on glucose-lowering medications. Without this lifestyle change to achieve T2D remission, the cost of Case 2′s illness might be conservatively estimated at approximately USD 9600 [76] per year for the next 25 years, totaling around, on average, an additional USD 240,000 in healthcare costs due to type 2 diabetes. Given the modest cost of the Kaiser program in comparison to potential costs of ongoing type 2 diabetes treatment, it would be beneficial for insurance companies to prioritize covering such programs to incentivize patients towards lifestyle change. Case 2 paid the cost (USD 4700) out-of-pocket, which is not a financially viable option for many patients.

These four cases illustrate not only the huge personal burden of chronic illness with respect to health, quality of life, and cost, but the enormous burden of chronic illness on the healthcare system. It must be noted, however, that a limitation of this case series is that all information was obtained from cases via self-report. Thus, cases were not always able to recall detailed information with respect to medical costs, spending, and insurance costs. LM approaches embody value-based care and present clear economic advantages, *although these advantages have yet to be rigorously measured and quantified*. Many medical guidelines begin by recommending lifestyle change as a critical component in the therapeutic approach [77,78,79]. Regretfully, emphasis is not largely placed on lifestyle changes in clinical practice, as clinicians are not trained or reimbursed for delivering LM. Simply put, while promoting a healthy lifestyle is a current standard of care recommendation, deploying LM in an appropriate therapeutic dose is not widely seen in Western medical practice. There is an urgent need for research demonstrating its value compared to and in combination with more widely applied treatment modalities for both health outcomes and cost savings. While current clinical comparative effectiveness research tends to focus on comparing medications and procedures to each other, this type of research would benefit from a study arm that includes lifestyle changes that are so often the foundation of clinical guidelines for non-communicable chronic diseases [79,80,81,82].

In order for LM to successfully be deployed into widespread clinical practice, healthcare practitioners must be able to identify published, peer-reviewed evidence of its advantages for health outcomes. This is foundational for evidence-based practice. Clinicians also need guidance documents based on this evidence. For insurance payers, health systems, and policymakers, the economic benefits of LM treatments must be quantified and compared to conventional disease management approaches; the development of this data is critical to support adoption of LM approaches because of the growing threat of healthcare expenditures.

Finally, individuals’ decisions to adopt healthy lifestyle behaviors can be better supported with information on consumer level cost savings, such as the reduced costs of medications taken, procedures avoided, and/or reduced frequency of medical appointments over time.

Thus, the following is a discussion of key research priorities needed to advance the field of LM by both characterizing benefits of LM treatments for health outcomes and examining potential beneficial economic sequelae. The contribution of each priority toward the quadruple aim is identified in italics.

(1) Conduct rigorously designed studies, including randomized controlled trials (RCTs) when appropriate [83], to adequately measure the effects of ITLC interventions on chronic disease health outcomes. Documenting the outcomes produced by ITLC interventions in peer-reviewed research is the first step in quantifying the economic benefits, thus building a case for incorporating LM into conventional medical practice and shaping standards of care. (*Improved population health; patient experience)*.

(2) Estimate cost and value of altered lifestyle expenses outside of medical care, and in comparison to medical care, including such tangible items as grocery costs or educational programs, as well as intangible factors, such as decreased absenteeism in the workplace and increased productivity, increased satisfaction with life, and better quality of life. These outcomes should be considered in future research in order to fully demonstrate the value of LM-based approaches. Other research may more directly examine the cost-effectiveness of programs, such as medically tailored meals, nutrition education and therapies, and work-site wellness programs aimed at treating the sickest segment of employees as opposed to typical worksite wellness programs targeting prevention. *(Reduced healthcare costs; patient experience)*.

(3) Measure effects of lifestyle medicine practice on providers’ burnout levels. The prevalence of provider burnout is widespread [84], yet LM providers report that engaging in LM practice has reduced their levels of burnout. Provider burnout is associated with increased medical errors [85], reduced safety [86] for patients, reduced productivity [87], and increased healthcare costs [88]. Improving the work experience of the healthcare worker can relieve burnout for the healthcare professional and improve patient satisfaction and health outcomes, which LM can address [58]. *(Provider well-being; reduced healthcare costs)*.

The final three proposed research priorities exclusively aim to better quantify the cost savings associated with ITLC, and such research could be leveraged to more clearly define the economic benefits of LM.

(4) Model the potential economic cost savings enabled by health improvements following lifestyle interventions as compared to usual disease progression and management. While characterizing medical costs is challenging, for some conditions, the typical healthcare spending on an annual basis was quantified [76], thereby making it possible to extrapolate potential savings for the patient and/or private health insurance or Medicare/Medicaid. For example, Case 1′s lifestyle intervention immediately avoided a costly gastric bypass surgery, which may be clearly quantified. However, the cost-savings of avoiding continued physician visits every 1–2 weeks, monthly appointments to aspirate fluid from his knees, multiple visits to the ER, and numerous medications, or the potential savings for Case 2 from avoided disease progression remain unclear. *(Reduced healthcare costs)*.

(5) Measure actual healthcare expenditures, such as those per member per month in a larger cohort of insured individuals, comparing those exposed to LM interventions vs. those who are not. The real goal for economic research on LM interventions needs to be the tracking of actual expenses and savings over time. One framework for examining these comparisons would be possible with leadership from an insurance provider to create a LM provider network, randomizing insured individuals to either the LM network or standard, non-LM network. Specific patient outcomes from among those who saw a provider over a defined time period could be compared. *(Reduced healthcare costs)*.

(6) Examine the effects of lifestyle medicine implementation upon different payment models. Whether Accountable Care Organizations, private pay, employer-sponsored healthcare, concierge, Medicare, Medicaid, or pay per service, there may be incentives and barriers to LM implementation that could be identified and quantified. Different payers have different needs, and services provided may have various impacts on their bottom line. *(Reduced healthcare costs)*.

## 5. Conclusions

This case series illustrates dramatic improvements in physical health and quality of life resulting from lifestyle change, particularly a transition to a WFPB diet. Research on LM interventions and clinical outcomes, economic impacts, comparative effectiveness, and effect sizes are emerging areas of study. Although case series provide weaker evidence than other study designs in the hierarchy of research evidence, these self-reported accounts provide a promising foundation to illustrate what may be possible for some individuals who engage in ITLC, justifying the research priorities outlined here to advance the quadruple aim and optimize health system performance. As the U.S. continues to battle higher healthcare costs, it is imperative that LM approaches are fully examined. While it is known that chronic diseases are an expensive problem, LM as a potential solution requires further study and examination. A fully informed discussion around the potential benefits and value of LM is emerging and will benefit from research as described here.

## Figures and Tables

**Table 1:** Self-reported prescription medication use for chronic disease pre- and post-lifestyle change ^1^.

	Pre-Lifestyle Change	Post Lifestyle Change
Case 1	Fentanyl, metoprolol, hydrocodone, felodipine, meloxicam, Neurontin, tramadol, atorvastatin, indomethacin, cyclobenzaprine, ranitidine, prednisone, zolpidem	None
Case 2	Metformin, endometriosis medication	No glucose-lowering medications, endometriosis medication only
Case 3	Ibuprofen, meloxicam, Flexeril, gabapentin, tramadol, cortisone injections, prescribed Vicodin but did not take it.	None
Case 4	Relpax, levothyroxine, losartan, potassium, angiotensin II receptor blockers (ARBs), triamterene, lisinopril, angiotensin-converting enzyme (ACE) inhibitor, Ceftin (cefuroxime), cephalexin, cefuroxime axetil, phenazopyridine, ciprofloxacin HCl, clarithromycin ER, amlodipine besylate, chlorthalidone, synthetic estradiol (estrace)	None

^1^ Medications are presented as reported by patients, thus both generic and brand names are included.

## References

[1] Leventhal R. (2020). The Value-Based Care Crossroad: Have ACOs Reached a Tipping Point?. https://www.hcinnovationgroup.com/policy-value-based-care/accountable-care-organizations-acos/article/21160230/the-valuebased-care-crossroad-have-acos-reached-a-tipping-point.

[2] Branning G., Vater M. (2016). Healthcare Spending: Plenty of Blame to Go Around. Am. Health Drug Benefits.

[3] Goodell S. (2020). Is an Accountable Care Organization Right for You?. https://www.webmd.com/health-insurance/types-of-health-insurance-plans.

[4] Matulis R. (2019). It’s Not Just Risk: Why the Shift to Value-Based Payment Is Also about Provider Flexibility—CHCS Blog. https://www.chcs.org/its-not-just-risk-why-the-shift-to-value-based-payment-is-also-about-provider-flexibility/.

[5] Jardim T.V., Mozaffarian D., Abrahams-Gessel S., Sy S., Lee Y., Liu J., Huang Y., Rehm C., Wilde P., Micha R. (2019). Cardiometabolic disease costs associated with suboptimal diet in the United States: A cost analysis based on a microsimulation model. PLoS Med..

[6] Kelly J.T., Su G., Zhang L., Qin X., Marshall S., González-Ortiz A., Clase C.M., Campbell K.L., Xu H., Carrero J.-J. (2021). Modifiable Lifestyle Factors for Primary Prevention of CKD: A Systematic Review and Meta-Analysis. J. Am. Soc. Nephrol..

[7] Colpani V., Baena C.P., Jaspers L., Van Dijk G.M., Farajzadegan Z., Dhana K., Tielemans M.J., Voortman T., Freak-Poli R., Veloso G.G.V. (2018). Lifestyle factors, cardiovascular disease and all-cause mortality in middle-aged and elderly women: A systematic review and meta-analysis. Eur. J. Epidemiol..

[8] Bellou V., Belbasis L., Tzoulaki I., Evangelou E. (2018). Risk factors for type 2 diabetes mellitus: An exposure-wide umbrella review of meta-analyses. PLoS ONE.

[9] Centers for Disease Control and Prevention How You Can Prevent Chronic Disease. https://www.cdc.gov/chronicdisease/about/prevent/index.htm.

[10] Centers for Disease Control and Prevention Health and Economic Costs of Chronic Diseases. https://www.cdc.gov/chronicdisease/about/costs/index.htm.

[11] Rowley W.R., Bezold C., Arikan Y., Byrne E., Krohe S. (2017). Diabetes 2030: Insights from Yesterday, Today, and Future Trends. Popul. Health Manag..

[12] Heidenreich P., Trogdon J.G., Khavjou O.A., Butler J., Dracup K., Ezekowitz M.D., Finkelstein E.A., Hong Y., Johnston S.C., Khera A. (2011). Forecasting the Future of Cardiovascular Disease in the United States. Circulation.

[13] Ward Z.J., Bleich S.N., Cradock A.L., Barrett J.L., Giles C.M., Flax C., Long M.W., Gortmaker S.L. (2019). Projected U.S. State-Level Prevalence of Adult Obesity and Severe Obesity. N. Engl. J. Med..

[14] George Washington School of Public Health (2021). Stop Obesity Alliance. Fast Facts: The Cost of Obesity. https://stop.publichealth.gwu.edu/sites/stop.publichealth.gwu.edu/files/documents/Fast%20Facts%20Cost%20of%20Obesity.pdf.

[15] Martin A.B., Hartman M., Lassman D., Catlin A., National Health Expenditure Accounts Team (2021). National Health Care Spending In 2019: Steady Growth for The Fourth Consecutive Year: Study examines national health care spending for 2019. Health Aff..

[16] Dobkin C., Finkelstein A., Kluender R., Notowidigdo M.J. (2018). Myth and Measurement—The Case of Medical Bankruptcies. N. Engl. J. Med..

[17] Himmelstein D.U., Lawless R.M., Thorne D., Foohey P., Woolhandler S. (2019). Medical Bankruptcy: Still Common Despite the Affordable Care Act. Am. J. Public Health.

[18] Caraballo C., Valero-Elizondo J., Khera R., Mahajan S., Grandhi G., Virani S.S., Mszar R., Krumholz H.M., Nasir K. (2020). Burden and Consequences of Financial Hardship from Medical Bills Among Nonelderly Adults with Diabetes Mellitus in the United States. Circ. Cardiovasc. Qual. Outcomes.

[19] American Diabetes Association (2018). Economic Costs of Diabetes in the U.S. in 2017. Diabetes Care.

[20] Khera R., Valero-Elizondo J., Okunrintemi V., Saxena A., Das S.R., De Lemos J.A., Krumholz H.M., Nasir K. (2018). Association of Out-of-Pocket Annual Health Expenditures with Financial Hardship in Low-Income Adults with Atherosclerotic Cardiovascular Disease in the United States. JAMA Cardiol..

[21] Cawley J., Meyerhoefer C., Biener A., Hammer M., Wintfeld N. (2015). Savings in Medical Expenditures Associated with Reductions in Body Mass Index Among US Adults with Obesity, by Diabetes Status. Pharmacoeconomics.

[22] McMacken M., Shah S. (2017). A plant-based diet for the prevention and treatment of type 2 diabetes. J. Geriatr. Cardiol..

[23] Esselstyn C., Golubic M. (2014). The Nutritional Reversal of Cardiovascular Disease-Fact or Fiction? Three Case Reports. Exp. Clin. Cardiol..

[24] Esselstyn C.B. (1999). Updating a 12-year experience with arrest and reversal therapy for coronary heart disease (an overdue requiem for palliative cardiology). Am. J. Cardiol..

[25] Barsotti G., Morelli E., Cupisti A., Meola M., Dani L., Giovannetti S. (1996). A Low-Nitrogen Low-Phosphorus Vegan Diet for Patients with Chronic Renal Failure. Nephron.

[26] Estruch R., Martinez-Gonzalez M.A., Corella D., Salas-Salvadó J., Ruiz-Gutiérrez V., Covas M.I., Fiol M., Gómez-Gracia E., López-Sabater M.C., Vinyoles E. (2006). Effects of a Mediterranean-Style Diet on Cardiovascular Risk Factors. Ann. Intern. Med..

[27] Appel L.J., Moore T.J., Obarzanek E., Vollmer W.M., Svetkey L.P., Sacks F.M., Bray G.A., Vogt T.M., Cutler J.A., Windhauser M.M. (1997). A Clinical Trial of the Effects of Dietary Patterns on Blood Pressure. N. Engl. J. Med..

[28] de Lorgeril M., Salen P., Martin J.-L., Mamelle N., Monjaud I., Touboul P., Delaye J. (1996). Effect of a Mediterranean type of diet on the rate of cardiovascular complications in patients with coronary artery disease insights into the cardioprotective effect of certain nutriments. J. Am. Coll. Cardiol..

[29] Tektonidis T.G., Åkesson A., Gigante B., Wolk A., Larsson S.C. (2015). A Mediterranean diet and risk of myocardial infarction, heart failure and stroke: A population-based cohort study. Atherosclerosis.

[30] Ornish D., Scherwitz L.W., Billings J.H., Gould K.L., Merritt T.A., Sparler S., Armstrong W.T., Ports T.A., Kirkeeide R.L., Hogeboom C. (1998). Intensive Lifestyle Changes for Reversal of Coronary Heart Disease. JAMA.

[31] Ornish D., Weidner G., Fair W.R., Marlin R., Pettengill E.B., Raisin C.J., Dunn-Emke S., Crutchfield L., Jacobs F.N., Barnard R.J. (2005). Intensive lifestyle changes may affect the progression of prostate cancer. J. Urol..

[32] Barnard N.D., Cohen J., Jenkins D.J.A., Turner-McGrievy G., Gloede L., Green A., Ferdowsian H. (2009). A low-fat vegan diet and a conventional diabetes diet in the treatment of type 2 diabetes: A randomized, controlled, 74-wk clinical trial. Am. J. Clin. Nutr..

[33] Burkitt D.P. (1982). Western diseases and their emergence related to diet. S. Afr. Med. J..

[34] Campbell T.C., Parpia B., Chen J. (1998). Diet, lifestyle, and the etiology of coronary artery disease: The Cornell China study. Am. J. Cardiol..

[35] Campbell T.C., Junshi C. (1994). Diet and chronic degenerative diseases: Perspectives from China. Am. J. Clin. Nutr..

[36] Tonstad S., Butler T., Yan R., Fraser G.E. (2009). Type of Vegetarian Diet, Body Weight, and Prevalence of Type 2 Diabetes. Diabetes Care.

[37] Wright N., Wilson L., Smith M., Duncan B., McHugh P. (2017). The BROAD study: A randomised controlled trial using a whole food plant-based diet in the community for obesity, ischaemic heart disease or diabetes. Nutr. Diabetes.

[38] Jakše B., Pinter S., Jakše B., Pajek M., Pajek J. (2017). Effects of an Ad Libitum Consumed Low-Fat Plant-Based Diet Supplemented with Plant-Based Meal Replacements on Body Composition Indices. BioMed Res. Int..

[39] McDougall J., Thomas L.E., McDougall C., Moloney G., Saul B., Finnell J.S., Richardson K., Petersen K.M. (2014). Effects of 7 days on an ad libitum low-fat vegan diet: The McDougall Program cohort. Nutr. J..

[40] Satija A., Bhupathiraju S.N., Rimm E.B., Spiegelman D., Chiuve S., Borgi L., Willett W.C., Manson J.E., Sun Q., Hu F.B. (2016). Plant-Based Dietary Patterns and Incidence of Type 2 Diabetes in US Men and Women: Results from Three Prospective Cohort Studies. PLoS Med..

[41] Turner-McGrievy G.M., Davidson C., Wingard E.E., Wilcox S., Frongillo E.A. (2015). Comparative effectiveness of plant-based diets for weight loss: A randomized controlled trial of five different diets. Nutrition.

[42] Mu M., Xu L.-F., Hu N., Wu J., Bai M.-J. (2017). Dietary Patterns and Overweight/Obesity: A Review Article. Iran. J. Public Health.

[43] Farmer B., Larson B.T., Fulgoni V.L., Rainville A.J., Liepa G.U. (2011). A Vegetarian Dietary Pattern as a Nutrient-Dense Approach to Weight Management: An Analysis of the National Health and Nutrition Examination Survey 1999–2004. J. Am. Diet. Assoc..

[44] Tuso P.J., Ismail M.H., Ha B.P., Bartolotto C. (2013). Nutritional update for physicians: Plant-based diets. Perm. J..

[45] Barnard N.D., Scialli A.R., Turner-McGrievy G., Lanou A.J., Glass J. (2005). The effects of a low-fat, plant-based dietary intervention on body weight, metabolism, and insulin sensitivity. Am. J. Med..

[46] Burke L.E., Styn M.A., Steenkiste A.R., Music E., Warziski M., Choo J. (2006). A randomized clinical trial testing treatment preference and two dietary options in behavioral weight management: Preliminary results of the impact of diet at 6 months—PREFER study. Obesity.

[47] American College of Lifestyle Medicine (2021). Plant-Based Diets and Weight Management. https://lifestyle-medicine.foleon.com/wfpb-nutrition/weight-management/intro/.

[48] American College of Lifestyle Medicine (2021). Plant-Based Diets and Type II Diabetes. https://lifestyle-medicine.foleon.com/wfpb-nutrition/type-ii-diabetes/intro/.

[49] American College of Lifestyle Medicine (2021). Plant-Based Diets and Cardiovascular Disease. https://lifestyle-medicine.foleon.com/wfpb-nutrition/cardiovascular-disease/intro/.

[50] Bogdanos D.P., Smyk D.S., Rigopoulou E.I., Mytilinaiou M.G., Heneghan M.A., Selmi C., Gershwin M.E. (2012). Twin studies in autoimmune disease: Genetics, gender and environment. J. Autoimmun..

[51] Cashman K.D., Shanahan F. (2003). Is nutrition an aetiological factor for inflammatory bowel disease?. Eur. J. Gastroenterol. Hepatol..

[52] Christ A., Lauterbach M., Latz E. (2019). Western Diet and the Immune System: An Inflammatory Connection. Immunity.

[53] Poutahidis T., Kleinewietfeld M., Smillie C., Levkovich T., Perrotta A., Bhela S., Varian B.J., Ibrahim Y.M., Lakritz J., Kearney S.M. (2013). Microbial Reprogramming Inhibits Western Diet-Associated Obesity. PLoS ONE.

[54] Procaccini C., Carbone F., Galgani M., La Rocca C., De Rosa V., Cassano S., Matarese G. (2011). Obesity and susceptibility to autoimmune diseases. Expert Rev. Clin. Immunol..

[55] Turner-McGrievy G.M., Wirth M.D., Shivappa N., Wingard E.E., Fayad R., Wilcox S., Frongillo E.A., Hébert J.R. (2015). Randomization to plant-based dietary approaches leads to larger short-term improvements in Dietary Inflammatory Index scores and macronutrient intake compared with diets that contain meat. Nutr. Res..

[56] Lifestyle Medicine. https://www.lifestylemedicine.org/ACLM/About/What_is_Lifestyle_Medicine_/Lifestyle_Medicine.aspx.

[57] Smith B. (2021). CMS Innovation Center at 10 Years—Progress and Lessons Learned. N. Engl. J. Med..

[58] Bodenheimer T., Sinsky C. (2014). From triple to quadruple aim: Care of the patient requires care of the provider. Ann. Fam. Med..

[59] Berwick D.M., Gilfillan R. (2021). Reinventing the Center for Medicare and Medicaid Innovation. JAMA.

[60] CostHelper (2021). How Much Does Gastric Bypass Surgery Cost?—CostHelper. https://health.costhelper.com/gastric-bypass-surgery.html.

[61] CostHelper (2021). How Much Does Sleep Apnea Treatment Cost?. https://health.costhelper.com/sleep-apnea.html.

[62] Healthgrades.com (2015). 5 Tips for Purchasing a TENS Unit: Healthgrades. https://www.healthgrades.com/right-care/bones-joints-and-muscles/5-tips-for-purchasing-a-tens-unit.

[63] Riddle M.C., Cefalu W.T., Evans P.H., Gerstein H.C., Nauck M.A., Oh W.K., Rothberg A.E., le Roux C.W., Rubino F., Schauer P. (2021). Consensus Report: Definition and Interpretation of Remission in Type 2 Diabetes. Diabetes Care.

[64] Medical News Today (2021). Does Medicare Cover Compression Stockings?. https://www.medicalnewstoday.com/articles/does-medicare-cover-compression-stockings#costs.

[65] CostHelper (2021). How Much Does Knee Replacement Cost?—CostHelper. https://health.costhelper.com/knee-replacement.html.

[66] Vodovotz Y., Barnard N., Hu F.B., Jakicic J., Lianov L., Loveland D., Buysse D., Szigethy E., Finkel T., Sowa G. (2020). Prioritized Research for the Prevention, Treatment, and Reversal of Chronic Disease: Recommendations from the Lifestyle Medicine Research Summit. Front. Med..

[67] Kahleova H., Dort S., Holubkov R., Barnard N.D. (2018). A Plant-Based High-Carbohydrate, Low-Fat Diet in Overweight Individuals in a 16-Week Randomized Clinical Trial: The Role of Carbohydrates. Nutrients.

[68] Leslie W.S., Ford I., Sattar N., Hollingsworth K.G., Adamson A., Sniehotta F.F., McCombie L., Brosnahan N., Ross H., Mathers J.C. (2016). The Diabetes Remission Clinical Trial (DiRECT): Protocol for a cluster randomised trial. BMC Fam. Pract..

[69] Karlsen M., Panigrahi G., Kelly J. (2021). 503-P: Intensive Lifestyle Interventions for Treatment towards Remission of Type 2 Diabetes: A Case Series. Diabetes.

[70] Barnard N.D., Cohen J., Jenkins D.J., Turner-McGrievy G., Gloede L., Jaster B., Seidl K., Green A.A., Talpers S. (2006). A Low-Fat Vegan Diet Improves Glycemic Control and Cardiovascular Risk Factors in a Randomized Clinical Trial in Individuals with Type 2 Diabetes. Diabetes Care.

[71] Esselstyn C.B. (2017). A plant-based diet and coronary artery disease: A mandate for effective therapy. J. Geriatr. Cardiol..

[72] Ornish D., Brown S.E., Scherwitz L.W., Billings J.H., Armstrong W.T., Ports T.A., McLanahan S.M., Kirkeeide R.L., Brand R.J., Gould K.L. (1990). Can lifestyle changes reverse coronary heart disease?: The Lifestyle Heart Trial. Lancet.

[73] Goldner B. (2019). Six-Week Raw, Vegan Nutrition Protocol Rapidly Reverses Lupus Nephritis: A Case Series. Int. J. Dis. Reversal Prev..

[74] Taylor R., Al-Mrabeh A., Sattar N. (2019). Understanding the mechanisms of reversal of type 2 diabetes. Lancet Diabetes Endocrinol..

[75] Kelly J., Karlsen M., Steinke G. (2020). Type 2 Diabetes Remission and Lifestyle Medicine: A Position Statement From the American College of Lifestyle Medicine. Am. J. Lifestyle Med..

[76] American Diabetes Association (2021). The Cost of Diabetes. https://www.diabetes.org/resources/statistics/cost-diabetes.

[77] American Diabetes Association (2021). Facilitating Behavior Change and Well-being to Improve Health Outcomes: Standards of Medical Care in Diabetes-2021. Diabetes Care.

[78] U.S. Department of Veterans Affairs (2018). Guidelines for Cancer Prevention. https://www.nutrition.va.gov/docs/UpdatedPatientEd/Cancer_Prevention.pdf.

[79] Styne D.M., Arslanian S.A., Connor E.L., Farooqi S., Murad M.H., Silverstein J.H., Yanovski J. (2017). Pediatric Obesity—Assessment, Treatment, and Prevention: An Endocrine Society Clinical Practice Guideline. J. Clin. Endocrinol. Metab..

[80] Arnett D.K., Blumenthal R.S., Albert M.A., Buroker A.B., Goldberger Z.D., Hahn E.J., Himmelfarb C.D., Khera A., Lloyd-Jones D., McEvoy J.W. (2019). 2019 ACC/AHA Guideline on the Primary Prevention of Cardiovascular Disease: A Report of the American College of Cardiology/American Heart Association Task Force on Clinical Practice Guidelines. Circulation.

[81] Mechanick J.I., Garber A.J., Handelsman Y., Garvey W.T., Beir D.M., Bohannon N.J., Bray G.A., Bush M.A., Evans J.G., Hurley D.L. (2012). American Association of Clinical Endocrinologists’ Position Statement on Obesity and Obesity Medicine. Endocr. Pract..

[82] Leroith D., Biessels G.J., Braithwaite S.S., Casanueva F.F., Draznin B., Halter J.B., Hirsch I.B., McDonnell M., Molitch M.E.E., Murad M.H. (2019). Treatment of Diabetes in Older Adults: An Endocrine Society Clinical Practice Guideline. J. Clin. Endocrinol. Metab..

[83] Katz D.L., Karlsen M.C., Chung M., Shams-White M.M., Green L.W., Fielding J., Saito A., Willett W. (2019). Hierarchies of evidence applied to lifestyle Medicine (HEALM): Introduction of a strength-of-evidence approach based on a methodological systematic review. BMC Med. Res. Methodol..

[84] Rotenstein L.S., Torre M., Ramos M.A., Rosales R.C., Guille C., Sen S., Mata D.A. (2018). Prevalence of Burnout Among Physicians. JAMA.

[85] Shanafelt T.D., Balch C.M., Bechamps G., Russell T., Dyrbye L., Satele D., Collicott P., Novotny P.J., Sloan J., Freischlag J. (2010). Burnout and Medical Errors Among American Surgeons. Ann. Surg..

[86] Salyers M.P., Bonfils K.A., Luther L., Firmin R.L., White D.A., Adams E.L., Rollins A.L. (2017). The Relationship Between Professional Burnout and Quality and Safety in Healthcare: A Meta-Analysis. J. Gen. Intern. Med..

[87] Shanafelt T.D., Mungo M., Schmitgen J., Storz K.A., Reeves D., Hayes S.N., Sloan J.A., Swensen S.J., Buskirk S.J. (2016). Longitudinal Study Evaluating the Association Between Physician Burnout and Changes in Professional Work Effort. Mayo Clin. Proc..

[88] Dyrbye L.N., Shanafelt T.D. (2011). Physician burnout: A potential threat to successful health care reform. JAMA.

